# Exploring Healthcare Paradoxes in Hospital Haemodialysis**—**A Qualitative Study

**DOI:** 10.1111/hex.70000

**Published:** 2024-08-30

**Authors:** Tone Andersen‐Hollekim, Torstein Hole, Marit Solbjør

**Affiliations:** ^1^ Department of Public Health and Nursing, Faculty of Medicine and Health Sciences Norwegian University of Science and Technology Trondheim Norway; ^2^ Medical Department, Ålesund Hospital, Møre og Romsdal Hospital Trust, Faculty of Medicine and Health Sciences Norwegian University of Science and Technology Trondheim Norway

**Keywords:** healthcare logics, healthcare paradoxes, hospital haemodialysis, patient–professional conflicts

## Abstract

**Introduction:**

The complex logics of healthcare systems inherit paradoxes that can lead to interpersonal conflicts impacting both patients and professionals. In this study, we aimed to identify and explore tensions and conflicts arising from paradoxes within hospital haemodialysis.

**Methods:**

We conducted a secondary supplementary analysis to previously collected qualitative data, including individual interviews with 11 patients and 10 nephrologists and focus groups involving a total of 13 haemodialysis nurses. Data were collected in Norway through three primary studies focused on exploring experiences of patient participation. For the current study, we employed thematic analysis.

**Results:**

Patient–professional conflicts emerged in three fundamental areas: (1) the hospital haemodialysis treatment, in which patients' views of treatment diverged from those of professionals, (2) patient–professional responsibility that became a negotiation point, with differing views on responsibilities, and (3) time, in which professional time took precedence over patients’ time, indirectly impacting patients due to resource allocation. These conflicts stemmed from paradoxes driven by unevenly validated principles, conflict of interest, and conceptual ambiguity.

**Conclusion:**

Altering healthcare logics by bringing in new perspectives or clarifying conceptual ambiguity could mitigate patient–professional conflicts. However, changing existing healthcare logics may give rise to new paradoxes and conflicts, which health services at various levels must address.

**Patient or Public Contribution:**

This secondary analysis utilized previously collected data from a project that did not involve patient or public contribution.

## 1 Introduction and Background

The term ‘paradox’, rooted in the Greek ‘para‐doxa’ (opposite opinion), signifies ‘contradictory yet interrelated elements that exist simultaneously and persist over time’ [1, p. 382]. Modern healthcare appears to be abundant with paradoxes [2]. A variety of these stem from the increasingly complex healthcare system with divergent and competing logics: socially constructed rules, practices and beliefs that uphold the institution [3]. These logics are enacted and intertwined at various levels of healthcare, impacting professional practice and patient care.

Paradoxes involve mixed messages that could engender tensions and conflicts among those involved [4]. Tensions are inherent in every human relationship and encompass several factors related to interpersonal relationships, including disagreements, misunderstandings, impaired trust and divergent expectations [5]. Conflicts transcend tensions, affecting those involved at a deeper and more personal level. In understanding interpersonal conflicts, we rely on the definition of Barki and Hartwick [6] focusing on three specific components, that is, negative emotions, disagreements and interference: ‘Interpersonal conflict is a dynamic process that occurs between interdependent parties as they experience negative emotional reactions to perceived disagreements and interference with the attainment of their goals’. According to Barki and Hartwick [6], an interpersonal conflict only exists when all the components are present. Unresolved conflicts pose a threat to the quality of care and patient safety [7], hinder productivity and increase employee turnover [8]. Both tensions and conflicts between patients and healthcare professionals are considered a pressing issue in today's healthcare, particularly in settings where the staff continuously interacts with patients and their families [9]. Kidney failure requiring dialysis is one such condition.

### 1.1 The Context of Hospital Haemodialysis

Kidney failure, defined as a glomerular filtration rate of less than 15 mL/min/1.73 m^2^, is a terminal condition that progresses from chronic kidney disease (CKD). Kidney failure necessitates long‐term healthcare for the patient, including dialysis [10]. Although traditional hospital haemodialysis is supplemented with home‐based dialysis in several countries, nearly 90% of patients worldwide in need of dialysis undergo hospital haemodialysis [11]. This treatment can be maintained for years, pending a kidney transplantation or as a life‐sustaining kidney substitute [10]. In accordance with international guidelines, hospital haemodialysis is administered by registered nurses on a regular basis, 3–4 days a week. The treatment aims to normalize vital parameters such as lab values and blood pressure according to standardized ideals of dialysis adequacy [12]. In addition, the treatment includes self‐management through adherence to food and fluid intake restrictions, medication and adaptation to the many physical and psychological manifestations of CKD [13].

Although hospital haemodialysis is designed to enhance patients’ physical well‐being, it is recognized that the treatment negatively impacts patients’ social life, psychological interests and health‐related quality of life [13]. Haemodialysis provides life support, but also restricts individuals’ lived experience of life [14]. Many patients strive to integrate treatment adaptation into their everyday life and find themselves confined within the schedules of dialysis. Entrusting their health and everyday life to healthcare professionals may lead to a loss of personal autonomy [15]. A significant number of patients on hospital haemodialysis are less satisfied with their treatment [16, 17], and suffer from anxiety [18] and depression [19]. However, individuals on hospital haemodialysis inevitably become familiar with institutional routines, fellow patients and renal staff, due to the recurrent treatment intervals. Over time, strong bonds form a ‘haemodialysis community’ [14]. For some individuals, the ‘family dynamic’ of this community represents a safety net, whereas for others, it represents restrictions and boundaries [20, 21].

### 1.2 Patient–Professional Conflicts in Haemodialysis Care

Regardless of the bonds associated with the haemodialysis community, tensions and conflicts between patients and providers exist and encompass a spectrum of situations. Previous research has associated conflicts with patient behaviour, that is, non‐adherence to treatment, in which patients do not follow recommendations for prescribed medical treatment, dialysis treatment or food and fluid restrictions [22]. This problem is widely acknowledged, with a worldwide prevalence of non‐adherence of more than 60% for both diet and fluid restrictions [23]. A survey by the American Kidney Fund [24] demonstrated that 36% of patients sign off from dialysis treatments early, whereas 18% of patients skip treatments. These behaviours will negatively affect patients’ health outcomes [25]. In a US review by Hashmi and Moss [26], conflicts arising from patient behaviour impacted the individual, fellow patients and staff. Terminating the dialysis session too early was associated with consequences only for the individual, whereas patients who arrived late for dialysis could disrupt the schedule for other patients. More extreme behaviour jeopardized the health and safety of others through verbal or physical abuse, along with threats to staff or other patients [22, 26, 27].

Several reasons have been suggested to explain patients’ behaviour, for example, lack of treatment motivation, typically manifested as patients not following medical advice or pushing to terminate the dialysis session ahead of time. Similarly, psychological problems associated with fear of death, loss of control, anxiety and depression lead to difficulties adhering to medical advice [26]. A more recent Australian review identified factors associated with socioeconomic issues, such as low education, disease‐related factors such as depression and treatment‐related factors such as length of dialysis sessions, to impact on patient adherence. Also, healthcare‐related factors such as inadequate training and engagement among providers as well as an overburdened health system influenced on patient adherence [28]. Regardless of the underlying cause, patient behaviour can pose a challenge to professional ethical principles. This is particularly true when healthcare professionals are tasked with promoting the best interests of the current patient, while simultaneously ensuring the welfare of other patients and their own interests [26].

Differences in patient–professional interests may lead to tensions and conflicts. Outcomes prioritized by professionals do not necessarily reflect CKD patients’ desired outcomes [29]. Increasing dialysis sessions from three to four times a week is associated with several beneficial outcomes for patients, such as increased survival and less need for fluid restriction. However, in a study from the United Kingdom, patients were willing to trade up to 2 years of life to avoid schedules of four sessions per week [30]. An Australian study found patients’ waiting times to cause conflicts with staff, for example, when patients were waiting to be connected to the dialysis machine, or experienced delays in the transport bringing them to their dialysis appointments [31].

The study conducted by Eppel and Marfisi [32] examined professional communication style as subject to patient–professional tensions and conflicts in CKD care. It was observed that professionals who showed defensive behaviours, such as avoidance, during unpleasant patient encounters often left patients feeling neglected or abandoned.

### 1.3 Healthcare Paradoxes Arising Conflicts

Rather than attributing conflicts solely to individual behaviour, it is possible to recognize that conflicts emerge from the coexistence of contradictory trends in healthcare. For example, on the one hand, there is individualization and person‐centred care, and on the other, there is standardization and production‐oriented systems. This dichotomy is manifested in several ways. Patients undergoing hospital haemodialysis are expected to meet self‐care requirements, which aligns with the perception of patients as responsible individuals capable of making informed decisions to achieve treatment goals. However, if patients fail to meet these expectations, they may face stigmatization [33, 34]. Not all patients are aware of the shifting trends in healthcare and can be confused when responsibilities traditionally held by healthcare professionals are transferred to them [35]. Healthcare professionals face contradictions when expected to comply with institutional standards of evidence‐based knowledge and care, while also accommodating patients’ preferences [36]. Pressures on healthcare organizations to achieve competing goals of lower cost and higher treatment quality pull professionals in opposite directions [37]. For patients, this could eventually lead to a decrease in the quality of care received [38].

### 1.4 Theorizing Paradoxes

Hofmann [2] distinguishes between three constructs of healthcare paradoxes: resolvable paradoxes, antinomies and aporias. The term ‘resolvable paradox’ is perceived as a paradox consisting of contradictory statements that can be reconciled, for example, contradictions stemming from ambiguous concepts, different interpretations of the healthcare system or conflicting interests. The term ‘antinomy’ denotes contradictions between two equally valid principles. Incorporating more profound principles of personal or professional values renders antinomies more challenging to resolve. The contradiction between standardizations and individualizations in healthcare is understood by Hofmann [2] as an antinomy. ‘Aporia’, the final of these paradoxes, is a ‘no way out’ paradox. Within aporias, the contradiction between fundamental principles leads to no resolution. Moreover, shifting perspective engenders new paradoxes. An example is the contradiction between the intention of doing good and the potentiality of doing harm in medicine [2]. The paradoxes should be understood as archetypes, thus not necessarily mutually exclusive. This implies that by scrutinizing the paradoxes from different perspectives, some paradoxes initially perceived as resolvable might transpire as antinomies, and some antinomies might be conceived of as aporias [2].

While recognizing that individual characteristics can both alleviate and exacerbate tensions and conflicts, the motivation for undertaking this study was to understand these within a wider context of the numerous paradoxes present in modern healthcare. Although both patient–professional conflicts in CKD care and healthcare paradoxes have been the subject of prior research, we found no studies that investigate these phenomena in relation to each other. Consequently, the objective of this study was to examine how healthcare paradoxes manifest as patient–professional tensions and conflicts within the context of hospital haemodialysis. Given that patients and healthcare professionals are positioned differently and therefore have distinct experiences and perceptions, we incorporated data from patients, haemodialysis nurses and nephrologists.

## 2 Material and Methods

### 2.1 Design

This study presents a secondary supplementary analysis [39] of data obtained from three previously conducted qualitative studies on the experiences of patients and healthcare professionals regarding patient participation in hospital haemodialysis. The first and second authors participated in conducting all three studies, while all authors of the present article took part in the second and third study. Data from the previous studies comprised focus groups with haemodialysis nurses and individual interviews with patients and nephrologists. Although the primary studies explored patient participation, the data also provided information on areas where patient–professional conflicts were present. This initiated our idea for a secondary analysis to further explore these areas, adding a supplementary analysis to the previously collected data.

### 2.2 Study Participants and Data Collection

In the primary studies, we applied a purposive sampling strategy [40] of study participants from Central Norway, including patients receiving hospital haemodialysis, and nurses and nephrologists working in haemodialysis units. Data were collected between 2015 and 2020. Patient data were collected in 2018 through individual in‐depth interviews comprising 11 patients (seven men, four women). The patients’ time on in‐centre haemodialysis varied from 6 months to 6 years. Eight patients were awaiting a kidney transplant when the interviews took place. Data from healthcare professionals were collected in 2015 and 2020 through three focus groups comprising 13 haemodialysis nurses (each group comprising four to five experienced nurses, all women) and individual interviews comprising 10 nephrologists, of whom four were women. Both focus groups and individual interviews were conducted utilizing semi‐structured interview guides, in which the questions were customized according to the study participants. We present themes from the interview guides summarized in Table 1.

**Table 1:** Resumé of the questions posed in the primary studies.

Patients' interview guide	How would you describe living with kidney failure and dialysis treatment?
	How are you involved in your treatment on a daily basis?
	How do you experience being seen and heard by the staff members?
Nurses' interview guide	How do you practice person‐centred care?
	How are the patients involved in their treatment?
	What challenges do you experience in patient participation?
Physicians' interview guide	How do you prepare your patients for dialysis?
	How can patients undergoing hospital haemodialysis participate?
	How do you promote patient participation in your clinical work?

### 2.3 Ethics

The Norwegian Agency for Shared Services in Education and Research granted permission for the primary studies (No. 40336, 59530 and 702797). Study participants voluntarily agreed to participate and signed an informed consent form before attending the primary studies. The aim of the present study was an extension of the primary studies, with all data anonymized. In line with the Norwegian Agency for Shared Services in Education and Research, we thus considered that the secondary analysis did not require a second ethical approval.

### 2.4 Analysis

In a supplementary analysis, a deeper exploration of an emergent issue or aspect of the data not addressed or only partially addressed in the primary study is undertaken [39]. Our secondary analysis followed the six steps of thematic analysis suggested by Braun and Clarke [41]. The data sets provided the foundation for coding and theme development. However, Braun and Clarke [41] highlight the importance of utilizing theory and concepts in the analysis process. In our analysis, we thus drew on the concepts of patient–professional tensions and conflicts and explored these through the theoretical lens of healthcare paradoxes, in line with the study's objective.

All three data sets were included in the secondary analysis. The process of familiarization was divided into two stages. All authors had previously participated in some or all the data analyses. For this study, each author read and coded one of the three data sets. Codes were developed based on text excerpts that highlighted events with the potential to create tensions, or explicit conflicts as described by the participants. As tensions and conflicts were selected as ‘sensitizing’ concepts, our analysis was centred on critically examining areas where these concepts were evident. Therefore, although all data sets were considered, not all segments were of interest. To develop themes, researchers met to compare data sets, review the initial coding and discuss the construction of preliminary themes. We looked for patterns and diversity and sought to explore overt and implicit meanings in the data sets [41]. Revising of themes was done as a process where the first author grouped codes into preliminary themes across the data sets and suggested themes, followed by several rounds of discussion and refining of themes where all authors participated.

When presenting the results, we use verbatim extracts from patients, haemodialysis nurses and nephrologists to illustrate our findings. To strengthen the transparency of our findings, we present additional quotes from study participants in Table 2. Eventually, after writing up themes, we explored these in relation to Hofmann's [2] healthcare paradoxes, identifying paradoxes that tensions and conflicts potentially built on. These are presented and debated in the discussion section.

**Table 2:** Additional quotes.

Theme	Quote	Participant
*Optimal treatment as a subject of negotiation*	*I'm a person who sets standards, and it seems to be tough on the nurses, especially those who've been here for many years. I noticed it from day one. At first, I had a lot of respect… but if I'm not respected, it's not so easy to give respect back.*	Patient 1
	*Some patients are better at fitting into the system.*	Nurse, Focus group 3
	*We do try to have regular conversations about how important it is to receive the treatment…we do have some who, um…who come late and leave early…and when you're going to be transplanted, it's important to follow up on the treatment we give you…there are those who are a bit too used to running their own lives.*	Physician 5
*Negotiating responsibilities*	*There's just so much they ask of me. I've got my diabetes to deal with, and then this dialysis thing on top of that. It's always ‘don't do this, don't do that…’ That's probably the hardest part. The pressure. But the doctors, they just come in and tell you what you must and mustn't do. I think they should think more about how it feels for us patients to be treated like this.*	Patient 2
	*We've got this one guy who really needs to step up his game. I gave him a bit of a lecture today. I reckon it's good for the patients when we set expectations for them.*	Nurse, Focus group 2
	*A lot of patients sort of step back and just want us to take care of them… like, ‘Oh, I guess I'm a bit heavy today, but you'll just have to deal with it’.*	Nurse, Focus group 1
*Hierarchizing time and limited resources*	*Well, for starters, there are more patients now…and we're short on space…we have to consider all the patients…and the taxi system…it's really frustrating for the patients when the taxis don't show up, or they show up late…and those shared taxis…it gives them a much worse dialysis experience.*	Physician 1
	*When you're in a rush, you usually just focus on the main stuff…like blood pressure, getting rid of extra fluid, and meds…and then the softer, less urgent parts of treatment tend to get overlooked.*	Physician 4
	*I just can't deal with arguing with them. Then they throw it back at me that they're swamped… but I reckon they should just bring in an extra nurse… especially when they have so many patients in the afternoon that things get hectic. It's all about keeping us safe, right?*	Patient 8

## 3 Results

Our analysis identified several areas within the haemodialysis community that were subject to tensions and conflicts between patients and healthcare professionals. Tensions and conflicts arose from divergent perspectives on haemodialysis treatment, patients’ self‐care responsibility and time management. We present our results through three main themes: (1) optimal treatment as a subject of negotiation, (2) negotiating responsibilities and (3) hierarchizing time and limited resources.

### 3.1 Optimal Treatment as a Subject of Negotiation

The execution of treatment was a primary source of tensions and conflicts between patients and healthcare professionals. For nurses and physicians, their practice was based on an understanding of the benefits of treatment, where more dialysis was considered superior to less dialysis. Following medical guidelines, they underscored and advocated patient adherence to the prescribed hours of dialysis. However, patients, through their lived experience, had learned the number of hours necessary for their well‐being. Their views on how and when the treatment should be administered could diverge from the views held by the professionals. Differences concerning the frequency of dialysis and the duration of each dialysis session resulted in negotiations between patients and professionals. Physicians spoke of ‘continuous trade‐offs’ (Physician 2), wherein they endeavoured to convince patients to accept more dialysis, while patients resisted spending more time at the hospital. Some physicians understood patient–professional tensions due to patients being thrust into a situation that they could not control. Consequently, patients could vent their frustrations upon healthcare professionals: ‘Not everyone is equally happy with us all the time, and we may have to accept that. They are upset because they have lost their health. And much more along the way’ (Physician 2).

Nurses interacted with patients daily, thus being explicitly engaged in everyday negotiations with them. The nurses were tasked with delivering treatment as prescribed by the physician. Although the prescription followed medical guidelines, nurses had discretion to assess each session individually and prolong treatment if they deemed it necessary. Their decisions were founded on measurable values, and less on patients’ wishes: ‘We hardly ever question the treatment a patient needs. It's just how we think; we base our decisions on the blood test results’ (Nurse, focus group 3). Excessive interdialytic weight gain unveiled patients’ non‐adherence to fluid restrictions in between dialysis sessions and was a common reason for nurses to prolong the sessions. However, some patients insisted on removing the maximum excess fluid within their pre‐scheduled treatment instead of prolonging the session. Others had experienced situations where nurses decided to draw more fluid than the patient found comfortable. Both scenarios could lead to tensions and conflicts. For nurses who were concerned about patient autonomy, professional responsibility put them in a dilemma when a patient refused to complete or prolong their dialysis session, or when patients’ views on treatment differed from medical guidelines. Being connected to the dialysis machine left patients in a vulnerable position, making negotiation challenging. Some patients experienced that their treatment time had been extended without involving them in decision‐making. ‘I was all set to wrap up the session, but then these two nurses came in and said I had to continue. I just couldn't take it anymore. I burst into tears’ (Patient 1).

This quote illustrates how conflicts could surface when patient autonomy contested professional expertise. Patients reported that their viewpoints about treatment were not entirely recognized by professionals. Decisions enacted without patient involvement left patients feeling ‘disregarded and powerless’ (Patient 11). Lack of dialogue with healthcare professionals was described by patients as an area of tensions and conflicts. Patients engaged with healthcare professionals in diverse manners. Some patients debated and negotiated frequency and techniques of treatment with professionals. They could yet experience the negotiation as an ‘unending struggle’ (Patient 7 and 11), depleting their vitality. Others refrained from broaching matters of personal significance out of respect for professional authority. Yet others conceded without further discussions upon realizing that professional opinions were prioritized over patient knowledge and autonomy within the logic of healthcare.

### 3.2 Negotiating Responsibilities

To attain optimal treatment, healthcare professionals advocated a collaborative approach, wherein patients bore the responsibility for adhering to food and fluid restrictions and following the prescribed medical treatment. Both nurses and physicians considered patient responsibility crucial for achieving optimal dialysis results. Some delegated the primary responsibility to patients: ‘It is the patients’ responsibility, not ours. We are here to assist them, but it is the patients’ responsibility. After all, they are the ones who are ill’ (Nurse, focus group 2). To encourage patients to assume responsibility for treatment, healthcare professionals prioritized informing and educating patients in self‐care and lifestyle modifications. If patients were reluctant towards assuming responsibility, it impacted on professionals’ capacity to fulfil their responsibilities. For example, if patients arrived at their dialysis session with excess fluid overload, indicating less adherence to fluid restrictions, this complicated the treatment and could eventually necessitate the nurses to prolong the session.

Physicians were concerned about how non‐adherent patients jeopardized their health. This concern led them to push for increased adherence in patients whom they perceived as lacking adequate self‐care. ‘We try to encourage them to do everything by the book, to get their blood test results in line with the treatment goals… so they don't get any more harm from the disease than necessary’ (Physician 4). Several patients felt burdened with excessive responsibility. They were torn between their lifestyle choices outside the hospital and adhering to treatment. These values frequently clashed. The patients felt that professionals focused on what happened inside the hospital and paid scant attention to patients’ everyday lives. They reported cascades of restrictions and requirements that undermined their autonomy and diminished their quality of life: ‘I am not allowed to eat phosphate, then I must compensate by taking these pills…No cheese. Not too much rice. Stay away from pasta. Don't drink too much [….] Lose weight’ (Patient 5). Some patients found the self‐care requirements unachievable and sought alternative ways to manage their lives as haemodialysis patients. To appear accountable within the healthcare logic, patients could abstain from food influencing their blood samples days before regular tests. However, nurses could sample blood randomly to detect irregularities in patients whom they considered non‐adherent. Hence, monitoring not only guided individual treatment but also became a means of assessing patients’ accountability. Patients who failed to assume the requisite responsibility, that is, adherence to medical advice, were perceived to arouse patient–professional conflicts: ‘There are some patients who don't want to do very much themselves. Of course, it is convenient to merely arrive here, recline, receive treatment, and return home. At home they eat cheese, drink milk… and when they're thirsty they drink, and then they get short of breath. And it's like ‐ ‘It's not my fault, I'm not involved in this, after all I attend every dialysis session’ (Physician 8). This quote suggests that some patients either resist conforming to professional expectations of collaboration or that they fail to do so. Both scenarios could indicate a belief among patients that the primary responsibility for their health and well‐being lies within the healthcare services.

### 3.3 Hierarchizing Time and Limited Resources

Becoming dependent on hospital haemodialysis necessitated a profound transformation in patients’ lives, which, governed by the clock, revolved around the dialysis sessions. Spending numerous hours each week on dialysis, preparing for dialysis and travelling to and from the hospital, ‘waiting’ emerged as a central concept in the patients’ narratives. Patients awaited taxis, awaited treatment to be initiated and completed, awaited to consult the physician. Repeating this pattern every other day for months and years, the way they allocated their time became of significant importance. None of the patients wanted to spend more time at the hospital than necessary. However, patients’ priorities for time did not necessarily align with the hospital's logic of time.

For healthcare professionals, time was associated with staff resources, which implied that when staff resources were limited, professionals were less capable of prioritizing patients' time. Both nurses and physicians described working days with a major focus on service production. Some compared the dialysis ward to a fast‐paced assembly line, on which patients were rolled in and out. ‘We don't have enough resources. I think it's all about doing things more efficiently. And it seems like everything needs to be done all at the same time’ (Nurse, focus group 2). Patients wanting to have their dialysis session executed as swiftly as possible and without complications put extra pressure on the nurses. However, from the professional perspective, patients had available time, whereas the professionals were short of time. Although both nurses and physicians emphasized time as a valuable factor in patient–professional encounters, and aimed to accommodate individual needs, time shortage limited their efforts. For example, if patients wanted their dialysis session at a different time than initially planned, this could disrupt the next shift of patients and was often not feasible. Additionally, limited material resources such as dialysis machines and chairs made it difficult to accommodate individual requests. Patients thus had to adapt to the pace of the clinic. This shows how productivity superseded individual considerations, and suggests that within the logic of healthcare services, different actors’ time stands in a hierarchical relation.

The time hierarchy incited conflicts and had several consequences for patients’ experiences of treatment. First, when subjected to waiting, patients experienced that their time was of lesser value than professional time. ‘We're always left waiting. It's like our time doesn't matter to anyone’ (Patient 10). A second consequence was that a shortage of professional time could lead to patients being rushed into dialysis upon arrival. Although some patients preferred to be put on dialysis as quickly as possible, others needed time to prepare, and being rushed disrupted this need. A third consequence was that despite professional focus on dialysis frequency, treatment sessions could be postponed, for example, if nurses failed to cannulate the patient's fistula. Being rushed into treatment represented a stressor for those needing time to prepare for their dialysis session. Postponing dialysis schedules could have a serious impact on patients’ well‐being, and eventually threaten their safety, as symptoms of kidney failure usually suppressed by regular treatment increased.

## 4 Discussion

The primary objective of this research was to explore the manifestation of healthcare paradoxes as tensions and conflicts between patients and professionals within the context of hospital haemodialysis. We accomplished this by identifying areas of tension and conflict between patients undergoing hospital haemodialysis and the healthcare professionals in these units. Three key areas were identified where tensions or conflicts were significant: the process of hospital haemodialysis treatment, the shared responsibility between patients and professionals regarding the treatment, and the issue of time, where professional time took precedence over that of the patients. Each of these areas involved negative emotions, disagreements and interference [6], which substantiates the existence of interpersonal conflicts. Our findings are discussed through the perspective of Hofmann's [2] healthcare paradox theory, which includes three types of paradoxes: resolvable paradoxes, antinomies and aporias.

### 4.1 Antinomies

Antinomies represent a contradiction between principles of equal validity, such as standardizations and individualizations in healthcare [2]. This became evident in our study through the mutual negotiation of treatment and responsibility between patients and professionals. Both nurses and physicians in our study advocated for patient involvement to achieve optimal treatment outcomes. However, optimal outcomes were predefined by biomedical standardizations directing treatment. The standardization was preferred over individualized care that incorporated patient preferences. Furthermore, when tensions arose due to disparities between medical interests and patients' personal interests, professional concerns were prioritized over patient concerns, which were not considered equally valid. The paradigm of person‐centred care recognizes that the patient brings a personal context and integrates this into medical expertise and the best available research [42]. Our findings suggest that this may not always be the case within haemodialysis care. Although not aligned with the ideals, this highlights the paradox inherent in person‐centred care: patients seek medical expertise to address their health problems, and hospitals are designed to meet medical needs. Therefore, there is an asymmetry rooted in the medical enterprise: professionals are present for a reason. Underestimating professional expertise may put patients’ health at risk [35, 43]. From this perspective, the power of the healthcare professional and the vulnerability of the patient could even be understood as an insolvable paradox, an aporia.

The hierarchization of time can be interpreted as another antinomy. Although the time of patients and professionals should be considered equally important, the prevailing emphasis on productivity in modern healthcare forces professionals to prioritize tasks necessary for production, or administrative tasks that do not directly involve patients [37]. Consequently, the patient's time is being compromised. However, patients could be preoccupied with their own needs, leading to limited awareness of the numerous professional decisions required within a hospital ward. This was demonstrated in the study by Burns and Smyth [31], where early‐arriving patients were frustrated about being placed on dialysis after late‐arriving patients but lacked the full understanding of the professional assessment underlying this decision. Ultimately, as shown in our study, delaying dialysis treatment due to a lack of time or staff resources can impact a patient's condition and increase the likelihood of hospitalization and even death [20].

Addressing antinomies may necessitate new perspectives or paradigms in healthcare [2]. The dominant model in healthcare has been the biomedical reductionist model, which attributes illness to a malfunction within a specific body part that can be treated [44]. An additional understanding could be the biopsychosocial model, which integrates the biological, psychological and social dimensions of illness [44, 45]. The biopsychosocial model supports person‐centred care [46] and is built on a tripartite concept of evidence‐based medicine, encompassing the best available research, clinical expertise and patient experience [42]. However, although biomedicine evaluates patients’ health through standardized measurements, the lack of measurability within the biopsychosocial model could hinder its implementation in the resource‐controlling political and managerial field [44]. Nonetheless, increasing focus on the patient as a person will provide a more integrated approach with the potential to contribute to the sustainability of health services [44]. This is supported by a growing acceptance of personalized care, including individualizing dialysis adequacy, thus moving away from the ‘one size fits all’ approach [12]. Although sufficient time and resources are mutually important for patients and healthcare professionals, its distribution is a concern for institutional management and relies on policymakers to ensure sound working conditions for professionals and optimize patient safety.

### 4.2 Resolvable Paradoxes

Hofmann's [2] concept of ‘resolvable paradoxes’ encompasses contradictory statements such as conflicting interests, ambiguous concepts or differing interpretations of the healthcare system. In this study, healthcare professionals emphasized the role of patients in ‘co‐producing’ health. This emerged as a source of negotiation and appeared to create a conflict of interest [2]. Transferring responsibility traditionally held by healthcare professionals to patients involves trusting patients to be accountable by making sound health choices [33]. However, this requires an adaptation to a behaviour defined by professionals. If patients fail to adapt, they risk being stigmatized by professionals as not being fully responsible [33, 34], as demonstrated in our study. The transfer of responsibility to patients implies a reduction in professionals’ workloads or the freeing up of time for other tasks. Within the context of production‐oriented healthcare, this could be a method of streamlining services. However, it is crucial to balance responsibility to avoid abandoning patients, that is, by compelling them to take on a responsibility for which they feel unqualified [43].

Concepts such as ‘self‐care’, ‘patient participation’ and ‘co‐production of health’ are ideals of modern healthcare, but these concepts are ambiguous and lack a clear understanding among both patients and professionals [47]. If patients’ understanding of the mission of healthcare services diverges from professional understandings, conflicts can arise. Some healthcare professionals in our study perceived patients as unwilling to take on the necessary self‐care responsibility, whereas some patients felt that this responsibility was impossible to fulfil. Although many patients appreciate being involved in issues related to their own health, others may find this role overwhelming or confusing [35]. In the biomedical model, the responsibility for patients’ health lies within the services. Thus, when attending dialysis sessions, patients may view their responsibility as fulfilled. That said, healthcare professionals, especially physicians, are educated within the biomedical paradigm and influenced by this thinking. Nurses are trained within a holistic perspective advocating for the patient perspective. However, as seen in our study, long‐term haemodialysis nurses may have adopted the biomedical model in their professional practice, focusing on laboratory values and dialysis technology [48]. Consequently, patient‐centred care suffers. Although a collaborative approach to achieving optimal treatment results was favoured by professionals in our study, patients’ collaborative role was limited to medical adherence. This highlights the paradox within patient participation: patients are encouraged to take on an active role, but their behaviour must comply with professional recommendations.

Drawing on Hofmann [2], we propose that conflict of interest is a paradox that could be resolved by identifying divergent interests and reaching a consensus, for instance, through shared decision‐making. Sharing decisions about a patient's health with the patient supports patient autonomy [49], brings to light patient values and preferences [50] and enhances patients’ involvement in treatment [51]. Shared decision‐making is applicable in self‐management education, medication issues and lifestyle changes [52], all of which are central areas in hospital haemodialysis. By highlighting patient preferences, shared decision‐making could also contribute to resolving antinomies. Acknowledging the complexity of the system and reconciling interpretations could assist in resolving stakeholders’ divergent interpretations of the healthcare system. Redefining or clarifying ambiguity within healthcare terms would contribute to resolving issues related to such ambiguity, including patient participation [2].

### 4.3 Aporias

Although our data identified few or no aporias, this does not imply that aporias do not exist within the context of hospital haemodialysis. According to Hofmann [2], the intention of doing good and the potential for causing harm in medicine is a candidate for an aporia. This is an overarching paradox within healthcare, including hospital haemodialysis, for example, when initiating dialysis treatment, which can sometimes harm the patient despite the intention being to save life. In accordance with Hofmann [2], we propose that paradoxes that initially appear as, for example, antinomies, may be understood as aporias upon closer examination.

Prior research has primarily focused on patient behaviour as subject to tensions and conflicts between patients and professionals [22, 26, 27, 28]. The present study extends beyond patient behaviour, demonstrating how the paradoxical logics of healthcare drive patient–professional tensions and conflicts. Altering healthcare logics involves stakeholders at various levels of health services, including policymakers. Some progress has already been made. As argued by Torreggiani et al. [12], customized care that includes patient preferences and values is increasingly idealized, and thus, customization could be understood as the new standardization. That said, changing healthcare logics could create new paradoxes and thus new conflicts, which health services at various levels must be prepared to discuss and resolve. However, some healthcare paradoxes are unavoidable and must be acknowledged and accepted [2].

## 5 Methodological Considerations

This study is the first to investigate paradoxes between patients and healthcare professionals in hospital haemodialysis, with data collected from three participant groups: nurses, physicians, and patients. Using both focus groups and individual interviews enabled us to explore conflicts between patients and professionals. The secondary analysis of the three data sets provided an opportunity to identify themes not sufficiently explored in the primary studies, making the reuse of data a sustainable approach prior to conducting a new study.

Field acquaintance represents a potential risk concerning ‘field blindness’. In this study, the first author had previously served as a haemodialysis nurse. By including a medical doctor and a sociologist in the research team, we ensured researcher triangulation [53], which enhances the credibility of the study as the phenomenon is examined through diverse lenses.

Data were collected in 2015, 2018 and 2020. The temporal aspect could be a limitation as the healthcare logics might have evolved. However, data collection with healthcare professionals yielded similar experiences despite the years between (2015 and 2020), indicating that the healthcare logics has not been radically altered. Given that the healthcare logics explored in this study are similar in other westernized countries, our findings should be of interest to both national and international hospital haemodialysis communities.

## 6 Conclusion and Implications

Our study demonstrates how tensions and conflicts between patients and healthcare professionals in hospital haemodialysis are rooted in paradoxes directing the patient–professional encounter. These paradoxes are associated with healthcare logics that challenge both patient and professional roles within modern healthcare. Altering healthcare logics by introducing new perspectives or clarifying conceptual ambiguity could mitigate conflicts arising from healthcare paradoxes. This responsibility lies with stakeholders at various levels of health services. Ultimately, some healthcare paradoxes must be acknowledged and accepted.

This study has significant implications for clinical practice. The regimen of treatment, self‐care requirements and time management instigated conflicts between patients and professionals, necessitating attention from clinicians and policymakers. The conventional dogma of ‘thrice a week’ dialysis is structured to cater to economic interests and facilitate the institution's daily routines [12]. A tailored approach that aligns with the individual's life beyond the ward could diminish self‐care requirements and alleviate conflicts emerging from the ‘one size fits all’ standard. Home‐based treatment possesses this advantage and has yet to fully realize its potential. Operating under contradictory approaches such as standardization and individualization pulls healthcare professionals in divergent directions, impedes person‐centred care and ultimately leads to conflicts between patients and professionals. Addressing this paradox requires engagement at the organizational and policy levels of healthcare. It is imperative that educational programmes incorporate modules to equip healthcare professionals with communication skills, conflict resolution strategies and understanding of power dynamics, while promoting person‐centred care and teamwork.

## Author Contributions

Tone Andersen‐Hollekim designed the study, collected the data, and drafted the manuscript. All authors contributed to the analysis, interpretation of the data, and rewriting the manuscript. All authors have given their final approval of the published manuscript.

## Conflicts of Interest

The authors declare no conflicts of interest.

## Data Availability

The data supporting the findings of this study can be made available upon reasonable request by contacting the corresponding author.
